# Neurological Presentations of COVID-19: Characteristic Features in a Case Series of Hospitalized Patients from Abu Dhabi, UAE

**DOI:** 10.1155/2021/5822259

**Published:** 2021-08-06

**Authors:** Asma Deeb, Palat Chirakkara Kumar, Nida Sakrani, Ravi Kumar Trehan, Vijay Ram Papinenei

**Affiliations:** ^1^Division of Paediatric Endocrinology, Sheikh Shakhbout Medical City, Abu Dhabi, UAE; ^2^Division of Neurology, Sheikh Shakhbout Medical City, Abu Dhabi, UAE; ^3^Division of Paediatric Rheumatology, Sheikh Shakhbout Medical City, Abu Dhabi, UAE; ^4^Division of Orthopaedic Surgery, Sheikh Shakhbout Medical City, Abu Dhabi, UAE; ^5^Radiology Department, Sheikh Shakhbout Medical City, Abu Dhabi, UAE

## Abstract

**Background:**

COVID-19 patients can present with neurological manifestations in the form of headache, dizziness, hyposmia, myalgia, peripheral neuropathy, acute cerebrovascular disease, and encephalopathy. Neurological involvement could be due to virus-induced brain hypoxia, brain infection, or immune reaction. We aim to describe the neurological presentation of COVID-19 patients and study their neuroimaging findings and disease outcome.

**Method:**

The study is a single-centre, retrospective, observational study in Sheikh Shakhbout Medical City (SSMC), Abu Dhabi, UAE. Patients diagnosed with COVID-19 between March and May 2020 who presented with neuropathological features with or without respiratory manifestations of COVID-19 were enrolled. Electronic records were studied for age, sex, duration of hospitalization, detailed neurological presentation, history or documented concomitant fever and respiratory features of COVID-19, inflammatory markers, neuroimaging, progress, and disease outcome.

**Results:**

Thirty-three patients of 10 nationalities presented with neurological manifestations. Mean (range) age was 51.4 (21–86) years. Twenty-four had comorbidities, and 18 had no prior or concomitant respiratory symptoms. Ten patients presented with encephalopathy and exhibited altered behavior/sensorium: 7 presented with myositis, 8 with stroke, and 4 with seizures, and 4 had peripheral and cranial nerve involvement. The mean (average) duration of hospital stay was 11.4 days (1-38) with the longest observed in stroke patients. Fifteen patients (45%) died and 3 (9%) had residual weakness. Serum ferritin, CRP, and procalcitonin were higher in the severe disease group and correlated with risk of death. Twelve of 22 brain images showed abnormalities including haemorrhage, infarcts, small vessel ischemia, and oedema. Risk of death was higher in older age but did not differ based on the underlying neuropathology.

**Conclusion:**

COVID-19 patients who present with neurological involvement have a higher risk of mortality which is aggravated by older age and higher inflammatory markers. The type of neurological pathology does not seem to influence the risk of mortality.

## 1. Background

Corona viruses are not primarily neurotropic viruses. They preferentially affect the respiratory system as their primary target. Angiotensin-converting enzyme-2 receptor (ACE 2) is the target for virus attachment which triggers the cascade of virus replication and further immune reaction. However, ACE 2 receptors are also found in glial cells in brain and spinal neurons; hence, it can attach and damage the neurological tissue and leads to stroke [1]. During the epidemic of SARS-CoV-1 in 2002, neurological involvement leading to various clinical presentations was recognized [2]. Many patients present with neurological symptoms such as headache, dizziness, hyposmia, myalgia, and neuropathy. A patient can also show features of neurology complications including encephalopathy, acute muscle injury, acute cerebrovascular disease, and a varying degree of impaired consciousness [3, 4].

Multiple mechanisms have been postulated to explain the neurological involvement in COVID-19. Severe pneumonia can result in systemic hypoxia and brain injury. Hypoxia, hypercarbia, anaerobic metabolism, and toxic compounds accumulation can result in neuronal swelling and brain oedema leading to neurological damage [5]. Apart from hypoxia, there is evidence that the intense immune reaction due to cytokine storms can lead to end organ damage.

A postulated mechanism for neurological involvement is through endothelial involvement leading to release of interleukin 6. This inflammatory reaction causes vascular leakage, activation of complement, and coagulation cascade leading to disseminated intravascular coagulation [6, 7]. Another mechanism is direct viral neuronal injury [8]. During the viremia phase of the illness, disruption of brain blood barrier occurs enabling a direct entry of the virus to the brain through the olfactory epithelium. In addition, the virus is shown to invade the peripheral nerve terminals and then enter the nervous system through the synapses [1].

Recognition of neurological involvement in severe respiratory disease like COVID-19 is crucial. Detailed neurological assessment and investigation can be challenging in patients with COVID-19 who are sick leading to underdiagnosis of the underlying pathophysiology. The challenge of obtaining MRI and neurophysiology investigations including EEG is compounded by the demand of safe nursing. Examining the cerebrospinal fluid when indicated might not be feasible considering the stretch on resources of infection control in the intensive care setting. In addition, controversy remains regarding the appropriate treatment measures like the use of high-dose steroids in patients with disseminated viral disease with lymphopenia. Another controversial example is the potential risks of using IVIG for Guillain-Barre syndrome (GCS) in patients with thrombotic risk factors.

## 2. Aim

We aim to describe the neurological presentation and course of hospitalized COVID-19 patients and study the neuroimaging findings. We also aim to assess factors influencing disease course and if the neurological pathology correlates with disease course/prognosis.

## 3. Method

The study is a single-centre, retrospective, observational study in Sheikh Shakhbout Medical City (SSMC), Abu Dhabi, UAE. We reviewed the clinical, radiological, and laboratory findings in patients diagnosed with COVID-19 who presented with neuropathological features with or without respiratory manifestations of COVID-19. Neurological presentation was considered based on the neurology consultation detailed in the patients' charts. The study period was during the first surge of the disease between March and the end of May 2020. The cases were identified through records tagged with the relevant international classification of diseases–10 (ICD–10) codes for COVID-19 and laboratory records of confirmed cases. The study was approved by the Central Department of Health Institution Research Board in Abu Dhabi and the SSMC local committee (MAFREC-192).

Patients who presented with neurological findings and were tested positive for COVID-19 were identified. Electronic records for these patients were studied for patients' age, sex, duration of hospitalization, detailed neurological presentation, history or documented concomitant fever and respiratory features of COVID-19, inflammatory markers, neuroimaging, progress, and disease outcome (recovered, had residual neurological disease, or are deceased).

Statistical analysis is done using the SPSS v.22. *P* value of <0.05 is considered significant.

## 4. Results: Case Series Description

Out of 1075 patients who were admitted with COVID-19 during the study period, we identified 33 patients who presented with neurological features and enrolled them in the study. All patients were confirmed to have COVID-19 based on a positive nasal-pharyngeal throat SARS-CoV-2 PCR test. 32 patients were males. Age ranged between 21 and 86 years with a mean age of 51.4 (median 52). 24 patients were South Asian (14 Indians, 9 Pakistanis, and 1 Afghani), 8 Arabs (2 Egyptians, 2 Emiratis, 1 Sudanese, 1 Syrian, 1 Iraqi, and 1 Yemeni), and 1 Filipino.

## 5. Comorbidities

12 patients (36%) have type 2 diabetes. Eight (24%) had hypertension, and 4 (12%) had ischemic heart disease. One patient had Rasmussen syndrome and was on antiepileptic medications, and another patient had Parkinson disease.

## 6. Presentation

The patients presented with a wide range of central and peripheral nerve systems features for a few hours to 10 days (mean 3.2 days) prior to presentation to the hospital. Eight had fever, and 7 had respiratory symptoms of cough and dyspnea. 18 had no prior or concomitant fever or respiratory symptoms on presentation.

The patients were divided into 5 categories based on their neurological features on presentation to our hospital. The categories were encephalopathy, myositis, seizures, stroke, and peripheral or cranial nerve injury (Table 1).

## 7. Neurological Categories

### 7.1. Encephalopathy (10 Patients)

In this category, patients presented with either altered behavior or sensorium. Three patients presented with behavioral changes. One presented with confusion and delirium of sudden onset. Another was confused with altered speech and hallucination, and the third presented with agitation. Seven patients presented with altered sensorium of various degree and mixture of symptoms. Symptoms were in the form of dizziness, headache, feeling light-headed, and vertigo. Four of them have complete loss of consciousness that varied from momentary to 15 minutes. All remained dizzy when they regained consciousness.

### 7.2. Myalgia/Myositis (7 Patients)

These patients presented with muscle ache either generalized [4] or affecting the lower limbs. Four had significant muscle tenderness over thighs with inability to walk. All patients had high creatinine kinase (CK) level. Mean (average) CK level was 3800 (388–17,153) with the normal lab range of 39–308. One patient had paroxysmal atrial fibrillation, and another had prolonged QT interval on ECG.

### 7.3. Stroke (8 Patients)

One patient presented with worsened weakness from a previous stroke associated with dysarthria. Three patients presented with right-sided weakness with one affecting the upper limb only. Four had weakness affecting the left side of the body. Two had associated dysarthria and fascial palsy.

### 7.4. Seizures (4 Patients)

These patients were the youngest amongst the group. One patient is a known patient with epilepsy (Rasmussen syndrome) but did not have any seizures for over a year. He presented with generalized tonic clonic convulsion. Three patients had convulsions for the first time. One had a focal seizure affecting the right arm and face. The other 2 had generalized convulsion. In one, seizures were uncontrolled and had to be intubated in the emergency department. Cerebrospinal fluid of these patients showed no abnormality.

### 7.5. Peripheral and Cranial Nerve Injuries

Two patients had Guillain-Barre syndrome. Both had lower limb weakness. One had finger tingling with sensory impairment, and the other had significant dysarthria and developed atrial fibrillation. The other 2 had cranial nerve involvement. One had dysarthria and nystagmus while the other had dysphagia and impaired gag reflex. The latter had a prior Parkinson disease and noted to have worsened tremors.

## 8. Duration of Hospitalization

All patients were admitted to the hospital. The mean (average) duration of hospital stay was 11.4 days (1-38). The duration of hospitalization varied amongst patients with different neurological diagnoses with the longest observed in those presenting with stroke. However, this was not statistically significant (*P* = 0.326) Table 1.

## 9. Inflammatory Markers

**Figure 1:**
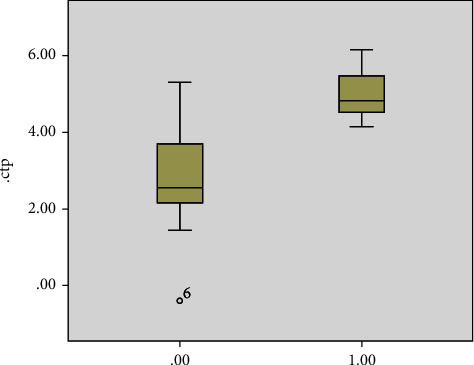
Outcome of disease of recovery or death based on age (0.00 recovery, 1.00 death).

**Figure 2:**
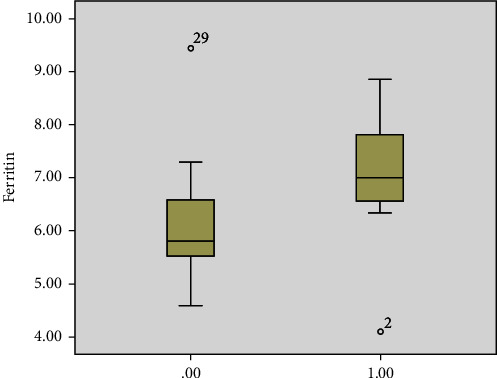
Median CRP based on the outcome of disease (0.00 recovery, 1.00 death).

**Figure 3:**
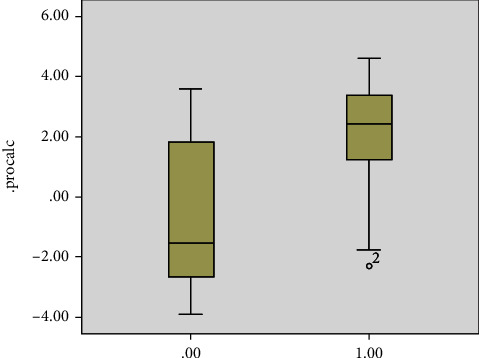
Median serum ferritin based on outcome of disease (0.00 recovery, 1.00 death).

Sixteen patients (48%) had leukocytosis on presentation, and 4 (12%) had leucopenia while 13 patients had normal white count. Thirty one (93%) patients had high CRP which correlated with the risk of death (*P* = 0.001) (Figure 1). 25 patients (75%) had a high ferritin level. The level did not vary significantly between the groups (Figure 2) but correlated with the risk of death (*P* = 0.04) (Figure 3). Similarly, serum procalcitonin correlates with death (*P* = 0.004) (Figure 3). None of the inflammatory markers showed a statistically significant difference between the groups.

## 10. Brain Imaging

**Figure 4:**
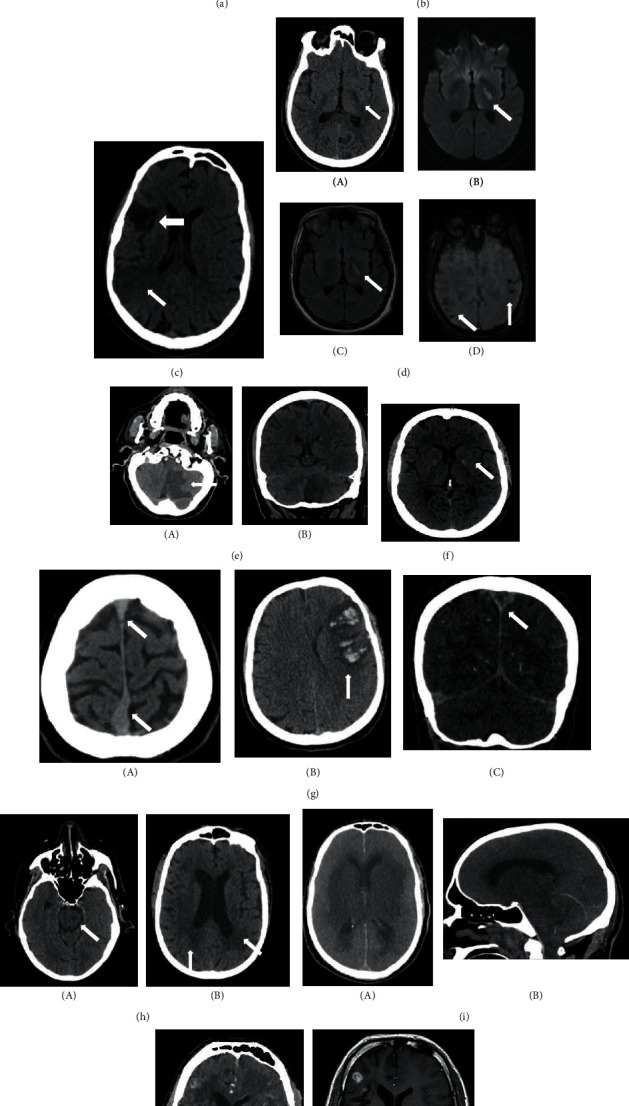
Brain imaging showing brain abnormalities in 10 patients (a–j). (a) Noncontrast CT head showing minor periventricular hypodensity suggestive of small vessel-related ischemia. (b) Noncontrast CT head showing multifocal white matter hyperdense foci suggestive of petechial haemorrhages (arrows pointing to areas of haemorrhage). (c) Noncontrast CT head showing old (big arrow) and evolving/new (small arrow) right MCA territory infracts. (d) (A) CT head showing low attenuation in the left internal capsule (B), (C) MRI showing left internal capsule high signal on diffusion weighted and FLAIR sequences, and (D) MRI gradient echo sequences showing blooming artifact suggesting blood break down products. (e) Axial and coronal non contrast CT head showing acute left cerebellar infract. (f) Noncontrast CT head showing a small area of low attenuation in the left internal capsule suggestive of a focal infract. (g) Noncontrast CT head showing (A) dense superior sagittal sinus (SSS) suggestive of venous sinus thrombosis, (B) haemorrhagic venous infract, and (C) CT venogram showing nonopacification of SSS (empty delta sign) due to venous sinus thrombosis. (h) Noncontrast CT head showing (A) lacunar infract left pons and (B) periventricular low attenuation changes in keeping with small vessel ischemia. (i) (A) Axial and sagittal noncontrast head CT showing marked sulcal effacement, extensive oedema, and loss of grey-white matter differentiation. (B) Sagittal section shows herniation of cerebellar tonsils. Appearances in keeping with severe cerebral oedema with coning. (j) Postcontrast CT (A) and MRI (B) of head showing focal enhancing lesion in the right frontal lobe.

22 patients had brain imaging of either CT, MRI, MRA, or combination. 10 scans were reported normal. There were 12 abnormal brain imaging (Figure 4, Table 2). Two scan images were not available through the hospital system due to technical difficulties. The abnormal findings were seen in 2 subjects from the altered sensorium group (periventricular small vessel ischemia and parenchymal haemorrhage; 7 from the stroke group showing infarcts (cerebral, internal capsule, cerebellar, venous haemorrhagic, and brain stem) and cerebral oedema; 1 from the seizure group with multiple areas of hyperintensities; and 2 from the cranial nerve palsy group showing involutional changes and subcortical white matter lesion).

## 11. Respiratory Support and Disease Outcome

**Figure 5:**
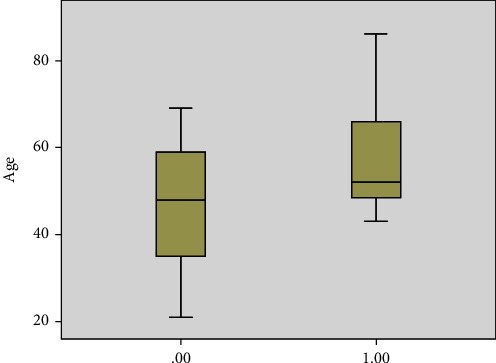
Level of procalcitonin based on outcome of disease (0.00 recovery, 1.00 death).

The outcome of the disease varied in the cohort. 15 patients died, 15 recovered, and 3 had residual neurological abnormalities (residual weakness). The patients who died were of the older age group (*P* = 0.021) (Figure 5). Of the 10 patients presenting with encephalopathy, 3 required artificial ventilation and all died. Two required high flow oxygen of whom one died. Five did not require respiratory support and all recovered. Six of the 7 patients presenting with myositis required artificial ventilation. One required high flow O_2_. All patients in this category died. Three patients of the stroke group died. Two of them required ventilation, and three required high flow oxygen. They had residual weakness on discharge. Two patients did not require respiratory support and recovered completely. All of those who presented with seizures recovered. One was ventilated for 1 day. Both patients who had Guillain-Barre syndrome recovered and did not require respiratory support. One patient with cranial neve palsy recovered, and the other one (who had an underlying Parkinson's disease) required high flow oxygen and died (Table 1).

## 12. Discussion

COVID-19 is known to affect the central and peripheral nervous system. The presenting neurological features related to COVID-19 could either be an effect of direct viral infection or inflammation of the nervous system and vasculature [9]. In a cohort of Ahmed et al., the most common central nervous symptoms reported are dizziness (16.8%) and headache (13.1%) [10]. Other central nervous system involvements include acute cerebrovascular disease, encephalopathy, neuralgia, encephalitis, ataxia, and seizures [3, 4, 11, 12]. Involvement of the peripheral nervous system can be manifested as reduced sense of taste or smell, skeletal muscle injury manifested as myositis, neuralgia, and Guillain-Barre syndrome [13]. Nervous system symptoms were significantly more common in severe cases of COVID-19 as compared with nonsevere cases (45.5% versus 30.2%) [10]. A severity score to predict inpatient mortality in COVID-19 patients was devised by Altschul et al. [14]. It has been shown that COVID-19 patients who have neurological deficits are at a higher risk of mortality. Eskandar et al. showed that altered mental status or stroke predicts a significantly higher risk of in-hospital mortality independent of disease severity [15].

In addition, up to 20% of COVID-19 patients who require intensive care unit admission had neurological manifestations of the disease [16, 17]. In our group, 12 (36%) required artificial ventilation and 15 (45%) died. In the cohort of Mao et al., most of the patients who presented with cerebrovascular disease and other neurological deficits had severe COVID-19 regardless of being associated with respiratory symptoms or not [3]. In this cohort, 2 out of six patients who presented with stroke did not have preceding or associated respiratory symptoms suggestive of COVID-19. Eighteen patients from our group had neither respiratory symptoms nor fever when presented with the neurological manifestations.

COVID-19 is known to be associated with a generalized thrombotic predisposition. This explains that stroke is seen as a common feature of neurology involvement. The hypercoagulability state associated with COVID-19 is likely to be a “sepsis-induced coagulopathy” and predispose to stroke [18]. The disease-related hypercoagulability state made the rationale of treatment with plasminogen activator for COVID-19-related stroke and low molecular weight heparinoids to reduce thrombosis [18]. Eight out of 33 (24%) patients with neurological manifestation in our group presented with stroke. All were treated with anticoagulants.

Patients who had a prothrombotic state, highly elevated D-dimer levels, and abnormal coagulation parameters have been shown to be associated with poor outcome [19]. Seven of the 8 patients who presented with stroke in our group had a high D-dimer. Studies showed that even young healthy individuals can present with stroke which can be large strokes [20]. Three of our patients who presented with stroke had no comorbidity or cardiovascular risk factor. Their age ranged between 38 and 55 years. Neurological complications, particularly stroke, can cause lifelong disability, resulting in long-term care requirement and significant health economic costs. Three patients in our cohort were discharged with residual weakness necessitating wheelchair use.

A retrospective study from China described the clinical characteristics of 113 COVID-19 patients of whom 20 had encephalopathy [16]. Mao et al. reported 14.8% of their 214 patients hospitalized with severe COVID-19 had impaired consciousness [3]. In our group, we had 7 patients (median age of 50) presenting with altered sensorium with various degrees of consciousness impairment (Table 1). Five had no fever or associated respiratory symptoms. All had a normal brain image and required no respiratory support except one who had parenchymal cerebral and cerebellar haemorrhage, required artificial ventilation, and died of severe disease (Table 2, patient 8, Figure 4(b)).

Various mechanisms for COVID-19 induced seizures have been suggested. Direct viral infection of neurons, strong inflammatory response, and entry of blood material into the brain resulting are plausible explanation for seizures and encephalopathy [2]. We had 4 patients presenting with seizure. In 3, seizures were of new onset. One patient had a seizure disorder (Rasmussen syndrome) controlled on anticonvulsion medications. Patients who presented with seizures were the youngest amongst the group presenting with neurological features. One required artificial ventilation, but all recovered and were discharged home.

Involvement of cranial and peripheral nerve deficits has been reported in COVID-19. An affected patient presented with various symptoms including anosmia, ageusia, areflexia, ophthalmoplegia, and fascicular oculomotor palsy and Guillain-Barre syndrome [13, 21]. We had 2 patients presenting with dysphagia, dysarthria, and nystagmus. These were diagnosed with cranial nerve palsy. One was 86 years old and had underlying Parkinson's disease which showed worsening of the tremors. He had a severe disease and died of respiratory failure. Another 2 patients presented with weakness and inability to move extremities. They were 35 and 55 years of age and diagnosed with Guillain-Barre syndrome. Both made a full recovery.

Muscle pain and elevated CK have been reported as manifestations of COVID-19 infection [22, 23]. Cases of rhabdomyolysis have also been reported [24]. Vasculitis or myositis has been suggested as etiology to muscle involvement in COVID [25]. Muscle involvement remained unclear if it is due to the direct effect of virus on the muscle tissue or an infection-mediated immune reaction resulting in elevated proinflammatory cytokines in serum causing the skeletal muscle damage. Mao et al. reported that 19.3% of severe COVID-19 and 4.8% of nonsevere cases had evidence of muscle injury [3]. They defined muscle injury as a combination of myalgia and elevated serum kinase level above 200 U/L. We had 7 patients presenting with muscle pain. Four of them had significant tenderness, particularly on the lower limbs. Their ages ranged from 45 to 60 years, and 2 of them had well-controlled type 2 diabetes. All patients in this group had severe COVID requiring ventilation, and all died. Their creatinine kinase on presentation ranged from 604 to 17135 (NR 39-308).

Various patterns for COVID-19 neurology abnormalities were reported in neuroimaging. The most frequently seen is medial temporal lobe, multifocal white matter hyperintense lesions with white matter microhaemorrhages [26]. Other radiological series have shown infarcts, microhaemorrhages, features of encephalopathy syndromes, and nerve root enhancement [27, 28]. We had 12 abnormal brain images out of the 22 scans done. There was a wide variation of imaging abnormalities in our group. These abnormalities included features of ischemia, cerebral and internal capsule infarcts, cerebral and cerebellar haemorrhages, cerebral oedema, and scattered white matter hyper intensity lesions (Table 2, Figures 4(a)–4(j)). It was reported that intracerebral haemorrhagic lesions were associated with worse clinical status [26]. The outcome of the disease in relation to the brain imaging was variable in our group (Table 2).

There is an overwhelming data in the literature about impact of age on disease outcome Multiple studies confirmed that the older the patient, the poorer the outcome and the higher the risk of mortality [29, 30]. In this study, the risk of death was significantly higher in older patients (Figure 1) regardless of the neurological presentation category.

Inflammatory markers have been shown to correlate with disease severity [14]. While we did not see any significant difference of CRP, ferritin, or procalcitonin between the different neurology groups, higher inflammatory markers were significant predictors of death (Figures 2, 3, 5).

The main limitation of our study is lack of extensive neurological investigations of these patients to pinpoint the exact underlying neurological pathology. The study was undertaken during the surge of COVID disease where the facilities were mainly directed to the intensive care aspects of COVID-19. Access to immediate neurological imaging was limited, and electrophysiology studies and cerebrospinal fluid examination were not immediately available due to infection control and safe nursing restrictions.

In conclusion, we have shown that COVID-19 can present with a variety of neurological manifestations. Similar to findings from the United States population, our patients presenting with neurological involvement have a higher risk of mortality which is aggravated by older age and higher inflammatory markers. The type of neurological pathology does not seem to influence the risk of mortality.

## Figures and Tables

**Table 1:** The table describes the neurology presentation category with the number of patients, age, comorbidity, duration of hospitalization, requirement for artificial ventilation, and disease outcome.

Neurology category		Number (out of 33)	Median age	Comorbidity	Mean (average) duration of hospitalization	Ventilation support	Disease outcome
Encephalopathy	Altered behaviour	**3**	63	1 DM	8 (6-12)	2	All died
				1 DM, HTN, IHD			
	Altered sensorium	**7**	50	1 HTN	10.2 (1-30)	1	6 recovered, 1 died
				1 DM, HTN, Dys			
				1 Dys			
				1 DM, HTN			
Myalgia/myositis		**7**	48	2 DM	6.8 (2-15)	6	All died
Stroke		**8**	57.5	4 DM	17.2 (5-38)	2	3 died, 3 had residual weakness, 2 recovered
				4 HTN			
				2 IHD			
				1 CHF			
Seizures		**4**	29	1 Rasmussen syndrome	10.25 (3-23)	1	All recovered
Peripheral & cranial nerve injury		**4**	59	1 DM	13.5 (4-29)	None	3 recovered, 1 died
				1 IHD, Parkinson's disease			

DM: diabetes mellitus; HTN: hypertension; IHD: ischemic heart disease; CHF: congestive heart failure; Dys: dyslipidemia.

**Table 2:** Brain imaging results on patients with different neurological presentation who are reported of having abnormal brain scans.

Patient number	Age	Presentation category	Disease outcome	Brain imaging	Figure
1	63	Altered behavior (delirium)	Died	Normal brain CT	
2	76	Altered behavior (confusion)	Died	Small vessel periventricular ischemia and atrophic changes in keeping with age	5.1
3	52	Altered behavior (agitation)	Died	Normal brain CT	
4	38	Altered sensorium	Recovered	Normal brain CT	
5	55	Altered sensorium	Recovered	Normal CT and MRI brain	
6	69	Altered sensorium	Recovered	Normal brain CT	
7	37	Altered sensorium	Recovered	Normal brain CT	
8	50	Altered sensorium	Died	Scattered parenchymal haemorrhage in cerebral and cerebellar hemispheres	5.2
9	60	Stroke	Residual weakness	CT: acute infarct in the right parietal lobe (right MCA territory)	5.3
10	52	Stroke	Residual weakness	CT: left internal capsule hypodensity indicating acute infarct	5.4
11	65	Stroke	Residual weakness	CT: large left sided cerebellar infarct with midline shift	5.5
12	53	Stroke	Recovered	CT: small focal hypodensity at the genu of the left internal capsule	5.6
13	38	Stroke	Recovered	CT: left frontal hemorrhagic venous infarct with moderate white matter oedema in the left frontal lobe	5.7/CSF
14	69	Stroke	Died	CT: pontine lacunar infarct	5.8
15	55	Stroke	Died	Marked generalized cerebral oedema with marked effacement of the surface sulci and basal cisterns and cerebellar tonsillar herniation. There is complete loss of definition of the basal ganglia and reduced grey-white matter differentiation	5.9
16	27	Seizures	Recovered	Normal brain CT	Normal CSF
17	44	Seizures	Recovered	Normal brain CT	Normal CSF
18	27	Seizures	Recovered	Normal brain CT	Normal CSF
19	31	Seizures	Recovered	White matter hyperintensity bifrontal and right occipital lobe	5.10/normal CSF
20	35	Peripheral/GBS	Recovered	Normal brain CT, MRI	Normal CSF
21	63	Cranial nerve palsy	Recovered	MRI brain: moderate involutional change with element of cerebellar and cerebral atrophy	Image not available
22	86	Cranial nerve palsy	Died	Two (right frontal and left frontal) nonspecific subcortical white matter lesions	Image not available

## Data Availability

Data is available on request.
